# Do patients with large vessel occlusion ischemic stroke harboring prestroke disability benefit from thrombectomy?

**DOI:** 10.1007/s00415-020-09882-5

**Published:** 2020-05-14

**Authors:** Alice Larsson, Camilla Karlsson, Alexandros Rentzos, Marcus Schumacher, Margareta Abrahamson, Arne Allardt, Anke Brederlau, Erik Ceder, Maria Davidson, Dennis Dunker, Thorsteinn Gunnarsson, Lukas Holmegaard, Mikael Jerndal, Jan-Erik Karlsson, Annika Nordanstig, Petra Redfors, Lars Rosengren, Turgut Tatlisumak, Katarina Jood

**Affiliations:** 1 Department of Clinical Neuroscience, Institute of Neuroscience and Physiology, The Sahlgrenska Academy at University of Gothenburg, Blå stråket 7, plan 3, 413 45 Göteborg, Sweden; 2 Department of Neurology, Sahlgrenska University Hospital, Göteborg, Sweden; 3 Department of Radiology, Institute of Clinical Sciences, The Sahlgrenska Academy at University of Gothenburg, Göteborg, Sweden; 4 Department of Diagnostic and Interventional Neuroradiology, Sahlgrenska University Hospital, Göteborg, Sweden

**Keywords:** Ischemic stroke, Large vessel occlusion, Thrombectomy, Acute care, Disability, Outcome, Mortality

## Abstract

**Objectives:**

Evidence of endovascular treatment (EVT) for acute large vessel occlusion (LVO) ischemic stroke in patients harboring substantial prestroke disability is lacking due to their exclusion from randomized trials. Here, we used routine care observational data to compare outcomes in patients with and without prestroke disability receiving EVT for LVO ischemic stroke.

**Methods:**

Consecutive patients undergoing EVT for acute LVO ischemic stroke at the Sahlgrenska University Hospital from January 1st, 2015 to March 31st, 2018 were registered in the Sahlgrenska Stroke Recanalization Registry. Pre- and poststroke functional levels were assessed by the modified Rankin Scale (mRS). Outcomes were recanalization rate (mTICI = 2b/3), symptomatic intracranial hemorrhage [sICH], complications during hospital stay, and return to prestroke functional level and mortality at 3 months.

**Results:**

Among 591 patients, 90 had prestroke disability (mRS ≥ 3). The latter group were older, more often female, had more comorbidities and higher NIHSS scores before intervention compared to patients without prestroke disability. Recanalization rates (80.0% vs 85.0%, *p* = 0.211), sICH (2.2% vs 6.3% *p* = 0.086) and the proportion of patients returning to prestroke functional level (22.7% vs 14.8% *p* = 0.062) did not significantly differ between those with and without prestroke disability. Patients with prestroke disability had higher complication rates during hospital stay (55.2% vs 40.1% *p* < 0.01) and mortality at 3 months (48.9% vs 24.3% *p* < 0.001).

**Conclusion:**

One of five with prestroke disability treated with thrombectomy for a LVO ischemic stroke returned to their prestroke functional level. However, compared to patients without prestroke disability, mortality at 3 months was higher.

## Introduction

Endovascular treatment (EVT) has become the gold standard for treating large vessel occlusion (LVO) ischemic stroke in the anterior circulation following the publication of several randomized trials showing it to be safe and effective [1–8]. However, the participants in these trials were highly selected and almost exclusively functionally independent prior to stroke. Thus, despite the clear evidence of the benefit of EVT in anterior circulation LVO, in clinical daily practice, stroke physicians are often faced with acute stroke patients for whom evidence-based data do not explicitly contribute to making treatment decisions.

Prestroke disability with functional dependency is relatively common among patients presenting with acute ischemic stroke, with a reported frequency between 13 and 19.5% [9–11]. Currently, there is no consensus on the use of EVT in this patient group. The American Heart Association guidelines [12] do not include LVO ischemic patients harboring prestroke dependency among those with indication for EVT, although the guideline states that the treatment may be reasonable, while the European Stroke Organisation does not mention prestroke disability in their guidelines on thrombectomy in acute ischemic stroke [13]. So far, data from observational thrombectomy registers are scarce. Recently, Goldhoorn et al. reported prestroke dependency in 11% of patients who underwent EVT for an anterior circulation LVO in the observational MR CLEAN registry [14]. Despite lower absolute frequency of favorable outcomes at 90 days in prestroke-dependent patients compared to prestroke-independent patients, about one in four reached their prestroke functional level after EVT, indicating benefit of the treatment.

In the present study, we used data from a prospective high-volume single-center registry to investigate outcomes of EVT in LVO ischemic stroke patients harboring prestroke disability. According to local practice, based on the assumption that the benefit of EVT is reasonable also in prestroke-dependent patients, the treating physicians did not routinely exclude this patient group from EVT. Thus, a relatively large proportion of patients harboring prestroke dependency underwent EVT. Here, we hypothesized that LVO ischemic stroke patients harboring prestroke dependency treated in clinical routine benefit from EVT and return to their prestroke functional level to a similar extent as prestroke-independent patients.

## Methods

This registry-based study included all patients receiving an arterial puncture with the intention of EVT for treating an acute large vessel occlusion ischemic stroke at the Sahlgrenska University Hospital from January 1st 2015 to March 31st 2018. The data were obtained from the Sahlgrenska Stroke Recanalization Registry which combines data from the Swedish Stroke register, The EndoVascular treatment in Acute Stroke registry (EVAS-registry), the Regional population registry and the patients’ medical records. Data on causes of death were obtained from the National Board of Health and Welfare.

The modified Rankin Scale (mRS) was used to define pre- and poststroke functional level. The prestroke mRS values were obtained from the patients’ medical records and the poststroke mRS values were calculated from the answers of the Swedish Stroke Registers’ questionnaire at 3 months, a method previously used and validated [15], and from data obtained from patients’ medical records. Prestroke disability was defined as mRS score ≥ 3. A favorable outcome was defined as returning to the prestroke functional level (mRS score) 3 months after the intervention.

Recanalization was estimated using the modified Thrombolysis in Cerebral Infarction score (mTICI). Achieved recanalization was defined as an mTICI score of 2b, 2c or 3. All patients were evaluated with National Institutes of Health Stroke Scale (NIHSS) immediately upon arrival. We defined early neurological improvement as a decrease of at least 4 points or a score of 0 on NIHSS 24 h after the intervention. Early neurological deterioration was defined as an increase of 4 points or more on NIHSS 24 h after the intervention. Complications accounted for in this study were symptomatic intracranial hemorrhage (sICH) and complications during hospital stay including pulmonary embolism, pneumonia, pulmonary edema, significant arrhythmia, epilepsy, urinary infections, kidney failure, intracranial hypertension, delirium, problems in the groin after puncture requiring treatment and malignant cerebral media artery infarction. SICH was defined according to the European Cooperative Acute Stroke Study III (ECASS III) criteria as having an intracranial hemorrhage (ICH) on radiologic follow-up combined with a clinical deterioration equal of 4 points or more on NIHSS 24 h after intervention, or death within 7 days [16].

Prespecified primary outcomes were return to at least the prestroke functional level and mortality 3 months after intervention. Secondary outcomes were recanalization, complications and early neurological improvement or deterioration.

### Statistical analyses

Differences in outcomes and baseline characteristics between patients with (mRS 3–5) and without (mRS 0–2) prestroke disability were investigated using the Mann–Whitney *U* test for continuous variables and Chi-squared or Fisher’s test for categorical variables. A binary logistic regression analysis was performed to investigate the associations between prestroke disability and return to at least the prestroke functional level as well as death at 3 months after intervention adjusted for the possible confounders’ age, sex, atrial fibrillation, stroke before index stroke, NIHSS score before intervention, recanalization, and complications during hospital stay. In this study, a two-tailed value of *p* < 0.05 was considered statistically significant. IBM SPSS version 25 was used for all statistical analyses.

## Results

**Fig. 1:**
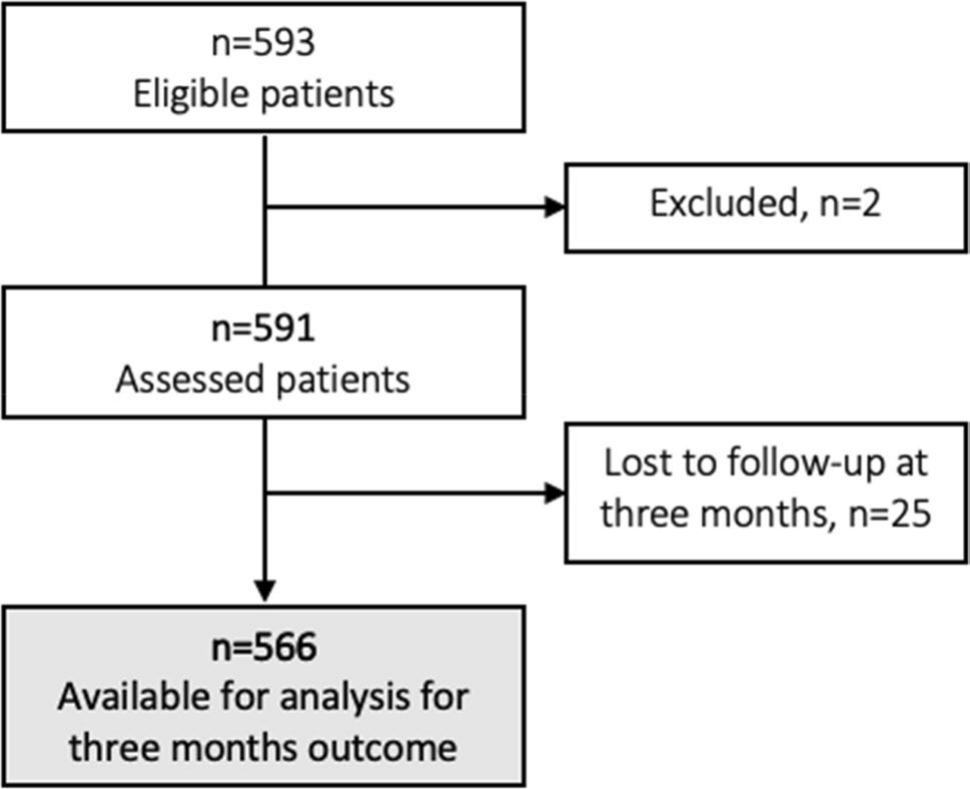
Flow chart of the study population. Two patients were underaged and were therefore excluded

From January 1st 2015 to March 31st 2018, 593 patients with anterior or posterior LVO ischemic stroke underwent EVT at the Sahlgrenska University Hospital. Two patients were younger than 18 years and were excluded. Of the remaining 591 patients, 90 had a prestroke disability (15.2%). Figure 1 illustrates a flowchart of the included patients and proportions of patients with missing data at 3 months follow-up.

Baseline characteristics for patients with and without prestroke disability are shown in Table 1. Patients with prestroke disability were older, more often women, and had more comorbidities such as atrial fibrillation and history of stroke compared to prestroke-independent patients. They also displayed higher NIHSS-scores before intervention. There were no significant differences in type of onset, the proportion of patients receiving IVT before intervention, nor vessel occlusion localization between the groups.

**Table 1:** Baseline characteristics

	Prestroke mRS ≥ 3 (*n* = 90)	Prestroke mRS 0–2 (*n* = 501)	*P* value
Age, median (range)	86 (57)	74 (82)	** < 0.001**
Women [*n*/*N* (%)]	54/90 (60.0)	218/501 (43.5)	** < 0.01**
Medical history			
Hypertension [*n*/*N* (%)]	65/88 (73.9)	322/496 (64.9)	0.102
Diabetes [*n*/*N* (%)]	20/90 (22.2)	84/499 (16.8)	0.217
Atrial fibrillation [*n*/*N* (%)]	56/90 (62.2)	204/499 (40.9)	** < 0.001**
Dyslipidemia [*n*/*N* (%)]	30/88 (34.1)	197/487 (40.5)	0.261
Stroke before index stroke [*n*/*N* (%)]	25/90 (27.8)	66/501 (13.2)	** < 0.001**
Onset characteristics			
Onset time			0.699
Confirmed [*n*/*N* (%)]	54/90 (60.0)	306/501 (61.1)	
Estimated [*n*/*N* (%)]	18/90 (20.0)	116/501 (23.2)	
Wake-up [*n*/*N* (%)]	17/90 (18.9)	72 /501(14.4)	
Unknown [*n*/*N* (%)]	1/90 (1.1)	7/501 (1.4)	
NIHSS before intervention—median (IQR)	18 (9)	16 (8)	** < 0.01**
IVT before intervention [*n* (%)]	38/90 (42.2)	252/500 (50.4)	0.153
Occluded artery			
Internal carotid artery [*n*/*N* (%)]	30/90 (33.3)	152/501 (30.3)	0.563
Middle cerebral artery [*n*/*N* (%)]	43/90 (47.8)	275/501 (54.8)	0.220
Anterior cerebral artery [*n*/*N* (%)]	1/90 (1.1)	17/501 (3.4)	0.247
Vertebral arteries [*n*/*N* (%)]	1/90 (1.1)	12/501 (2.4)	0.446
Basilar artery [*n*/*N* (%)]	8/90 (8.9)	50/501 (10.0)	0.753
Posterior cerebral artery [*n*/*N* (%)]	2/90 (2.2)	18/501 (3.6)	0.510
Spontaneously recanalized [*n*/*N* (%)]	9/90 (10.0)	51/501 (10.2)	0.959

*P* values below the threshold for statistical significance (p < 0.05) are given in bold

*mRS*  modified Rankin Scale, *IQR* interquartile range, *sICH*  symptomatic intracranial hemorrhage, *IVT* intravenous thrombolysis, *NIHSS* National Institutes of Health Stroke Scale

Differences between groups were investigated with the Mann–Whitney *U* test for continuous and the Chi-squared tests for categorical variables. Fisher’s test was used when any category comprised five patients or less. NIHSS before treatment was missing for 1 patient with prestroke mRS 3–5 and for 5 patients with prestroke mRS 0–2

### Early outcome

Prestroke-dependent and independent patients did not differ with respect to recanalization rate or time from groin puncture to recanalization (Table 2). NIHSS score at 24 h after the intervention was significantly higher among prestroke-dependent patients, and the proportion of patients with postintervention improvement of ≥ 4 points on the NIHSS was somewhat higher among prestroke-independent than prestroke-dependent patients (62.0 versus 54.4%, respectively), although this difference was not statistically significant (*p* = 0.179, Table 2). There were also no significant differences between the groups for early neurological deterioration, sICH, or in-hospital death, but prestroke-dependent patients were more often affected by complications during hospital stay compared to prestroke-independent patients (55.2% vs. 40.1%, *p* < 0.01).

**Table 2:** Outcomes

	Prestroke mRS ≥ 3 (*n* = 90)	Prestroke mRS 0–2 (*n* = 501)	*p* value
Early outcomes			
Recanalization rate [*n* (%)]	72/90 (80.0)	426/501 (85.0)	0.211
Groin puncture to recanalization, hh:mm, median (IQR)	00:45 (00:48)	00:52 (01:04)	0.174
Improvement on NIHSS ≥ 4 points or back to 0 [*n* (%)]	49/86 (57.0)	310/486 (63.7)	0.184
Deterioration on NIHSS ≥ 4 points [*n* (%)]	10/86 (11.6)	54/486 (11.1)	0.918
NIHSS 24 h after intervention, median (IQR)	13 (12)	8 (13)	** < 0.01**
In-hospital deaths [*n* (%)]	10/90 (11.2)	51/501 (10.2)	0.758
sICH [*n* (%)]	2/90 (2.2)	32/500 (6.4)	0.086
Complications during hospital stay [*n* (%)]	48/90 (55.2)	192/501 (40.1)	** < 0.01**
Outcomes 3 months after intervention			
Return to at least prestroke mRS [*n* (%)]	20/88 (22.7)	71/478(14.8)	0.062
Death [*n* (%)]	43/88 (48.9)	116/478 (24.3)	** < 0.001**

*P* values below the threshold for statistical significance (p < 0.05) are given in bold

*NIHSS* National Institutes of Health Stroke Scale, *sICH* symptomatic Intracranial Hemorrhage

Recanalization defined as mTICI score of 2b-3. Differences between groups were investigated with the Mann–Whitney U- test for continuous and the Chi-squared tests for categorical variables. Fisher’s test was used when any category comprised five patients or less. Time from groin puncture to recanalization was missing in 2 and 36 among patients with and without prestroke disability, respectively

### Functional outcome at 3 months

**Fig. 2:**
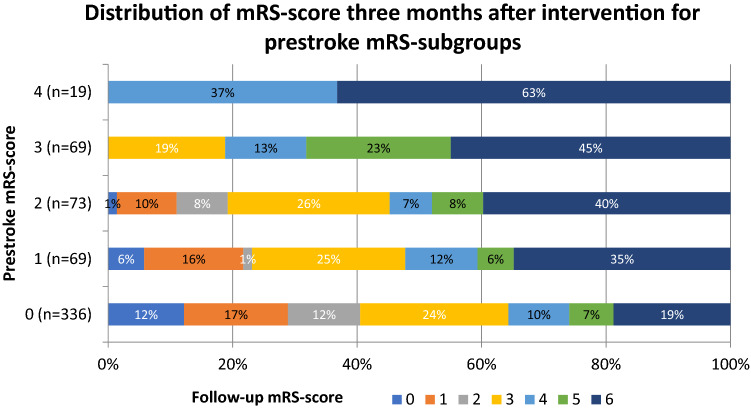
Distribution of mRS score 3 months after intervention for prestroke mRS subgroups. *mRS* modified Rankin Scale

Figure 2 shows the distribution of mRS scores 3 months after intervention for each prestroke mRS score. The proportion of patients returning to at least prestroke functional level did not differ significantly between prestroke-dependent and independent patients (22.7% vs. 14.8%, *p* = 0.062, Table 2). Reperfusion status was strongly associated with the chance for returning to prestroke functional level in both groups. None of the 17 patients with unsuccessful recanalization returned to prestroke functional level compared to 20 of 71 (28%) with successful recanalization among the prestroke-dependent (*p* < 0.01), and only 2/73 (2.7%) compared with 68/405 (17%) among prestroke-independent patients (*p* < 0.001). The strong association between successful recanalization and favorable outcome at 3 months remained in the multivariable regression model (Table 3). In this model, prestroke disability showed independent positive association, and NIHSS score before the intervention independent negative association with favorable outcome.

**Table 3:** Multivariable odds ratios and 95% confidence intervals of return to at least prestroke functional level at 3 months after intervention

	Odds ratio	CI lower	CI higher	*p* value
Age^a^	0.98	0.96	1.00	0.052
Female sex	1.12	0.69	1.83	0.643
Prestroke disability	2.64	1.34	5.19	** < 0.01**
Atrial fibrillation	1.18	0.70	1.98	0.540
Stroke before current stroke	1.17	0.60	2.30	0.649
NIHSS before intervention^b^	0.93	0.90	0.97	**0.001**
Successful recanalization	9.90	2.36	41.6	** < 0.01**
Complications during hospital stay	1.60	0.96	2.66	0.074

*P* values below the threshold for statistical significance (p < 0.05) are given in bold

*NIHSS* National Institutes of Health Stroke Scale, *CI* confidence interval

^a^Per 1 year increase

^b^Per one point increase in NIHSS score

### Mortality at 3 months

Three months after the intervention, patients with prestroke disability displayed significantly higher mortality compared to prestroke-independent patients (48.9% vs. 24.3%, *p* < 0.001, Table 2). Among patients with prestroke disability, mortality at 3 months did not differ between those with and without successful recanalization (46.2% vs 56.3%, *p* = 0.16).

The association between prestroke disability and mortality 3 months after intervention was further analyzed using multivariable logistic regression adjusting for the potential confounders’ age, sex, atrial fibrillation, history of stroke, NIHSS score before intervention, recanalization and complications during hospital admission. In this model, increasing age, prestroke disability, higher score of NIHSS before intervention, history of stroke, and unsuccessful recanalization were independently associated with a higher risk of death (Table 4), whereas no association between death and complications during hospital stay, or sex were observed.

**Table 4:** Multivariable odds ratios and 95% confidence intervals of death at 3 months after intervention

	Odds ratio	CI lower	CI higher	*p* value
Age^a^	1.03	1.01	1.05	** < 0.01**
Female sex	1.21	0.79	1.86	0.380
Prestroke disability	1.93	1.12	3.33	** < 0.05**
Atrial fibrillation	1.51	0.96	2.32	0.074
Stroke before current stroke	1.84	1.08	3.16	** < 0.05**
NIHSS before intervention^b^	1.10	1.06	1.14	** < 0.001**
No recanalization	3.29	1.94	5.56	** < 0.001**
Complications during hospital stay	1.48	0.97	2.24	0.069

*P* values below the threshold for statistical significance (p < 0.05) are given in bold

*NIHSS* National Institutes of Health Stroke Scale, *CI* confidence interval

^a^Per one year increase

^b^Per one point increase in NIHSS score

**Fig. 3:**
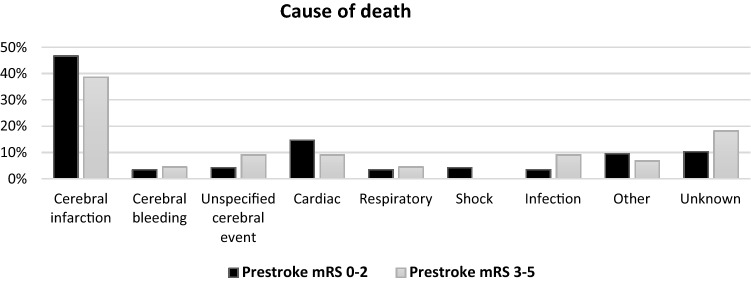
Cause of death for those who died within 3 months after intervention divided by prestroke mRS score. Total number of deaths 159 (*n* = 116 for prestroke mRS 0–2, n = 43 for prestroke mRS 3–5). *mRS* modified Rankin Scale

Figure 3 shows causes of death for patients with and without prestroke disability. Cerebrovascular disease was the most frequent cause of death followed by cardiac disease and infections, with no significant difference between prestroke-dependent and independent patients.

### Patients with non-stroke-related prestroke disability

Of 90 patients with prestroke disability, 25 had a prior clinical stroke and 65 had non-stroke related disability. The two groups did not differ with respect to NIHSS score before intervention (median score 18 and 19 in those with and without prior stroke, respectively, *p* = 0.725). Neither did they differ significantly with respect to recanalization rates (84% vs 78%), early neurological improvement (56% vs 54%), sICH (4% vs 1.5%), complications during hospital stay (44% vs 60%), return to at least prestroke functional level (28% vs 20%), nor mortality at 3 months (44% vs 49%), *p* > 0.183 all through.

## Discussion

Prestroke-dependent patients undergoing EVT for LVO ischemic stroke in clinical routine were older, had more comorbidities (atrial fibrillation and stroke before index stroke) and had more severe strokes (higher NIHSS before intervention) compared to prestroke-independent patients. Even so, recanalization rates, sICH, and the proportion of patients returning to their prestroke functional level were not significantly worse when compared to prestroke-independent patients. The findings that more than half of the patients with prestroke disability showed early neurological improvement and that one of five improved back to prestroke functional level 3 months after the intervention indicate a benefit of the treatment also in this patient group. However, complications during hospital stay were more common in the prestroke-dependent group, and mortality 3 months after the intervention was higher.

Outcomes of EVT in LVO ischemic stroke patients harboring prestroke disability were previously reported from two observational registries [14, 17]. The proportion of patients with prestroke disability in these registries were comparable to ours, with 157 of 1441 (11%) in the MR CLEAN registry [14] and 23 of 131 (17%) in a single-center study from Israel [17]. The present study adds another 90 patients to the previously systematically reported 180 patients with prestroke disability undergoing EVT for LVO ischemic stroke.

Similar to findings in our study, the previous reports from the two observational registries [14, 17] indicated beneficial effects of EVT in prestroke-dependent patients. In both studies, a substantial proportion (27% and 35%) of prestroke-dependent patients maintained their previous functional level at follow-up after 3 months. Moreover, early neurological improvement did not differ between prestroke-dependent and independent patients, and successful recanalization predicted a favorable outcome in both groups. Thus, results from our study are in concordance with the two previous real-life reports and further strengthens the evidence suggesting benefit of EVT in LVO ischemic stroke patients harboring prestroke disability. Taken together, the combined result from the three existing observational studies suggests that clinicians can be recommended to not routinely exclude patients harboring prestroke disability from EVT.

However, despite a substantial proportion of patients maintaining their prestroke functional level after EVT, our study and the two previous real-life reports show high mortality, up to 50%, 3 months after the intervention among patients harboring prestroke disability. We found that mortality at 3 months was independently associated with higher age, unsuccessful recanalization, comorbidities, stroke severity, and prestroke disability, whereas there was no increase in in-hospital death nor sICH among prestroke-dependent patients. Thus, the higher mortality in prestroke-dependent patients seems partly associated with the increased fragility related to older age and comorbidity. An important key question is how the positive effects of EVT with prevention of accumulated disability in prestroke-dependent patients stand against a relatively high short-term mortality. To better understand the balance between the beneficial effects and mortality after EVT in this particular patient group, both with respect to the individual and the society at large, further studies, including cost-effectivity analyses as well as more detailed measurements of disability and causes of death are required.

This study has some obvious limitations. First, it is not a randomized study, and all comparisons between prestroke-dependent and independent patients must be interpreted with caution as the two groups differ with respect to a number of essential characteristics. Most importantly, the difference in baseline mRS between the groups results in a non-equal definition of favorable outcome (return to prestroke functional level), as a shift between different steps of the scale do not correspond to the same size of change. Moreover, different methods were used to assess the pre- and poststroke functional levels, the use of mRS for assessing prestroke disability is less standardized compared to poststroke mRS and is probably more vulnerable to interrater variability. Lastly, we did not have information on the cause of prestroke disability and could therefore not identify patients with transient conditions contributing to the restricted functional level, such as surgery, bone fractures, infections or heart conditions. The strengths of the study include a relatively large series of consecutive patients undergoing EVT for LVO ischemic stroke in clinical routine which is reflected by the higher mean age and higher proportions of patients with previous strokes and comorbidities compared to the selected patients included in randomized studies [18]. We also included patients with posterior LVO ischemic stroke. Moreover, a relatively large proportion of patients had prestroke disability, and there were few missing data and low rates of loss to follow-up.

## Conclusion

About one-fifth of patients with prestroke disability and LVO treated with EVT returned to their prestroke functional level which is comparable to levels of patients without prestroke disability, suggesting that prestroke-dependent patients should not routinely be excluded from EVT. However, mortality at 3 months was higher, partly related to older age and increased fragility, which should be kept in mind when selecting patients for EVT in clinical practice. To better understand the balance between the beneficial effects and short-term mortality after EVT, further studies are required.
